# Improving survival rates of newborn infants in South Africa

**DOI:** 10.1186/1742-4755-2-4

**Published:** 2005-08-11

**Authors:** Robert Pattinson, David Woods, David Greenfield, Sithembiso Velaphi

**Affiliations:** 1 MRC Maternal and Infant Health Care Strategies Research Unit, Department of Obstetrics and Gynaecology, University of Pretoria, Pretoria, South Africa; 2 School of Child and Adolescent Health, Groote Schuur Hospital, University of Cape Town, Cape Town, South Africa; 3 Department of Paediatrics, Chris Hani Baragwanath Hospital, University of the Witwatersrand, Johannesburg, South Africa

## Abstract

**Background:**

The number, rates and causes of early neonatal deaths in South Africa were not known. Neither had modifiable factors associated with these deaths been previously documented. An audit of live born infants who died in the first week of life in the public service could help in planning strategies to reduce the early neonatal mortality rate.

**Methods:**

The number of live born infants weighing 1000 g or more, the number of these infants who die in the first week of life, the primary and final causes of these deaths, and the modifiable factors associated with them were collected over four years from 102 sites in South Africa as part of the Perinatal Problem Identification Programme.

**Results:**

The rate of death in the first week of life for infants weighing 1000 g or more was unacceptably high (8.7/1000), especially in rural areas (10.42/1000). Intrapartum hypoxia and preterm delivery are the main causes of death. Common modifiable factors included inadequate staffing and facilities, poor care in labour, poor neonatal resuscitation and basic care, and difficulties for patients in accessing health care.

**Conclusion:**

Practical, affordable and effective steps can be taken to reduce the number of infants who die in the first week of life in South Africa. These could also be implemented in other under resourced countries.

## Introduction

Of the approximately 130 million infants born worldwide each year, it is estimated that four million infants die during the first month of life [1]. The vast majority of these neonatal deaths occur in poor countries where standards of both maternal and newborn care are low. One of the Millenium Development Goals is to reduce the number of childhood deaths under the age of five years by two thirds from 95 per 1000 to 31 per 1000 by 2015 [2]. In South Africa, approximately 33% of deaths of under five-year-olds, 44% of infant deaths (before one year), and 87% of neonatal deaths (in the first month) occur during the first seven days after birth [3,4]. If the Millenium Development Goal of significantly reducing childhood deaths is to be achieved, a substantial reduction in early neonatal deaths will be required, especially in poor countries. The first steps in improving early neonatal survival are to document the number and rate of deaths during the first week, identify the common causes and look for modifiable factors. Only then can a logical approach be made to plan intervention strategies.

The early neonatal death rate in South Africa is not known but a survey in health facilities in the Cape Province about live-born infants weighing 1000 g or more showed a wide range of first week deaths, between 4.4 and 17.0/1000 in different regions with higher rates in rural areas [5]. The mortality rate during the first 7 days of life increased by an additional 62% if infants weighing between 500 and 999 g at birth were included [6]. Any attempt to record early neonatal deaths in South Africa would, therefore, have to take into account regional differences and birth weight categories. In order to address the challenge of improving pregnancy outcome, a facility-based audit of perinatal deaths in South Africa was started.

The Perinatal Problem Identification Programme (PPIP) was developed in the 1990s by the Research Unit for Maternal and Infant Health Care Strategies of the South African Medical Research Council (MRC) and has been extensively field tested since 1996 [7]. The aim of the Programme was to identify the common causes of death and associated factors which could be addressed to reduce the perinatal mortality rate. Basic perinatal birth data and causes of death are recorded. Both possible and probable modifiable factors are also noted. Probable factors could be directly linked to the death. Data from various sites can be analysed separately or together. Thus perinatal care indices (perinatal mortality rate/low birth weight rate), patterns of disease and modifiable factors can be combined for various groupings of sites, e.g. regional, provincial or national; or primary, secondary and tertiary levels of care; or metropolitan, city and town, and rural areas. Although not time consuming or labour intensive, PPIP relies on the presence of regular mortality meetings to discuss perinatal deaths and the possible shortcomings in care. As this requires personal commitment to manage the system, it unfortunately cannot yet be introduced at all sites where births occur in South Africa. Therefore, these data may present a best scenario as only sites with enthusiastic staff were available for inclusion.

The classification method used in PPIP to describe the causes of perinatal death was first used in Aberdeen by Sir Dugald Baird and colleagues in the 1940s [8]. This system clearly points to where prevention can be targeted. It was later modified and modernised by Whitfield et al in 1986 [9] and adapted by Pattinson et al in 1989 [10] for use in developing countries, and again in 1995 to include the concept of modifiable factors [11]. The definition of modifiable factors used in PPIP was adapted from the confidential enquiries into maternal deaths in the United Kingdom in 1985 [12]. The primary objective of PPIP is not to conduct an inclusive nationwide survey but to provide a tool to identify ways to improve the quality of perinatal care. With large numbers of deliveries, the major causes of perinatal deaths and their order of magnitude can be determined.

The aim of this paper is to examine the PPIP data-base in order to propose ways of reducing the mortality rate during the first week of life of live born infants weighing 1000 g or more at delivery. In developing countries with limited resources, these are the early neonatal deaths most likely to be avoidable.

## Methods

Data were collated from 102 sentinel sites within the public health service in South Africa. All perinatal deaths (stillbirths and neonatal deaths of 500 g or more) at these health care facilities were recorded over four years from 1^st ^October 1999 to 30^th ^September 2003. At each site all perinatal deaths were discussed at regular mortality audit meetings. While most first week deaths in infants weighing less than 2000 g at birth were captured, some deaths that occurred between the time of discharge from the health facility and 7 days of age in heavier infants may have been missed. Therefore, there may be an underestimation of deaths due to infections in infants weighing 2000 g or more. After review by the medical and nursing staff involved in the maternal and neonatal care, the probable primary and final causes of death were identified as well as any modifiable factors. The primary cause was defined as the underlying obstetric factor or condition which started a train of events that resulted in the death (why the death occurred, e.g. placental abruption) while the final cause was defined as the pathological process in the infant that actually caused the death (how the infant died, e.g. hypoxia). Primary causes identify pregnancy complications that can often be prevented (e.g. eclampsia) while final causes highlight areas of inadequate neonatal care (e.g. immaturity related deaths). The listed modifiable (avoidable) factors included missed opportunities for good care and examples of substandard care. These draw attention to areas of maternal and newborn care where improvements are needed.

The MRC unit contacted all services using PPIP and requested them to electronically send their data for collation, using PPIPWIN v2 (Simply Software^®^). This software package utilises a simple, user-friendly computer-based programme. Once basic perinatal data are entered, the programme calculates various perinatal care indices, describes the medical conditions that led to the perinatal deaths and lists modifiable factors associated with the deaths. Each site was categorised as metropolitan (the large amalgamated cities such as Cape Town), cities and towns, or rural areas. This categorisation was chosen as it grouped the hospitals and clinics into naturally comparable units, covered most of the institutional deliveries occurring in those areas and was thought to be more representative of population based data than any other combination. Most metropolitan areas are served by teaching hospitals and represent a fully functioning, tiered health care system, with all patients in the area having relatively easy access to tertiary care if needed. The city and town grouping represents areas where patients usually have easy access to primary and secondary level institutions, but where there is some difficulty in accessing tertiary hospitals. Finally the rural grouping represents primary care facilities, with the patients having to be referred for either secondary or tertiary care. Often health care facilities in cities and towns and rural areas had to provide levels of care beyond their means due to an inability to refer these patients. It was decided not to combine the data by levels of care across the country because of the very different referral patterns. All data were therefore categorised by site of delivery and not area of residence. The number of unrecorded deaths after home births is unknown, but is estimated not to be large. Only probable modifiable factors related to deaths are included in this analysis. Data on live born infants weighing 500 to 999 g were excluded as the reliability of these data were questionable. Some very small infants were coded as stillbirths in error, or their data were not recorded, as they were regarded as non-viable. Late neonatal deaths were also not considered as many infants are not closely followed by the health care services after the first week of life. Data were not available from the private sector where most of the community with health insurance receive care.

Infants with low-weight unrelated to maternal hypertension or any other identified obstetric cause were coded as "idiopathic SGA". Infection as a final cause of death included both prenatal infections (e.g. syphilis) and infections after delivery (e.g. necrotising enterocolitis).

Patient, administration and health worker related modifiable factors, which probably lead directly to the death, were considered. Sometimes more than one modifiable factor could be identified for a single death.

Ethics approval for the initial studies using PPIP was obtained from the University of Pretoria's Faculty of Health Sciences. The programme has since been taken over by the national and provincial Departments of Health and is approved by all health institutional Chief Executive Officers where it is used. Patient anonymity is assured at all times.

As this paper specifically addresses infant deaths in the first week of life, data on stillbirths have been excluded but are available on the PPIP website [7].

## Results

During the four year period, data on 452 927 live born infants weighing 1000 g or more were recorded from the 102 study sites (Table 1). These sites were grouped into metropolitan (200 738 infants), cities and towns (146 999 infants) and rural (105 190 infants). The low birth weight rates (percentages) at these sites were 15.1%, 15.5% and 11.4% respectively. While the annual number of live births in the public sector in South Africa is not known, it is estimated at 800 000. Therefore, the study sample consisted of approximately 20% of all live births in the public health service. The hospitals and clinics where these births took place may present the best scenario as they were the health centres willing and enthusiastic to join the project. Data were not available for the approximately 20% of births which occurred in the private sector.

**Table 1:** Number of live births by birth weight and place of birth in South Africa over four years 1999–2003

	**Metropolitan**	**Cities & Towns**	**Rural**	**All sites**
1000–1499 g	4068	2520	765	7353
1500–1999 g	7928	5204	2449	15581
2000–2499 g	18353	15073	8743	42169
2500 g+	170389	124202	93233	387824

Total	200738	146999	105190	452927

During the study period, 3916 live born infants, who weighed 1000 g or more and died in the first seven days, were identified. A further 17 were excluded as they were stillbirths incorrectly categorised as early neonatal deaths. The most common primary causes of death were "spontaneous preterm birth" (35.1%) and "intrapartum asphyxia and birth trauma" (33.4%) while the most common final causes of death were "hypoxia" (35.3%) and "immaturity related" (35.1%). Most of the deaths due to "hypoxia" (1187) were in infants weighing 2000 g or more. Mechanical trauma was uncommonly coded as a final cause of death, suggesting that the primary cause of "intrapartum asphyxia and trauma" reflected mainly foetal hypoxia in labour. There were more deaths without identified causes in rural than other areas.

The number, percentage and rates of primary and final causes of early neonatal deaths in the three regions are given in Table 2 and Table 3 respectively. The rates (per 1000 live births) for "intrapartum asphyxia and birth trauma" as a primary cause of death were highest in city and town (2.99) and rural (3.75) areas where death rates associated with "spontaneous preterm birth" were 4.33 and 4.0 respectively.

"Immaturity related" rates as a final cause of death were lowest in metropolitan areas (1.61) and higher in city and town (4.37) and rural (3.88) areas. "Hypoxia" rates were also higher in city and town (3.5) and rural (3.85) areas than metropolitan areas (2.3). Mortality rates due to infection would probably have been higher if late neonatal deaths were also considered.

**Table 2:** Primary causes of death in the three geographical groups (number, percentage and rate/1000 live births).

**Primary Causes**									
	**Metropolitan**			**Cities and Towns**			**Rural**		
	**Number**	**%**	**Rate**	**Number**	**%**	**Rate**	**Number**	**%**	**Rate**

Spontaneous preterm birth	324	25.47	1.61	636	40.64	4.33	421	38.41	4.00
Intrapartum asphyxia/birth trauma	362	28.46	1.80	439	28.05	2.99	394	35.95	3.75
Congenital abnormality	169	13.29	0.84	123	7.86	0.84	59	5.38	0.56
Hypertensive disorders	114	8.96	0.57	109	6.96	0.74	43	3.92	0.41
Infections	77	6.05	0.38	109	6.96	0.74	43	3.92	0.41
Abruptio placentae	89	7.00	0.44	53	3.39	0.36	23	2.10	0.22
Idiopathic SGA	46	3.62	0.23	25	1.60	0.17	14	1.28	0.13
Other antepartum haemorrhage	13	1.02	0.06	17	1.09	0.12	16	1.46	0.15
Pre-existing maternal disease	15	1.18	0.07	9	0.58	0.06	9	0.82	0.09
Other	58	4.95	0.31	35	2.88	0.31	72	6.75	0.70
Misclassified intra-uterine death	5			10			2		

Total	1272	100.00	6.34	1565	100.00	10.65	1096	100.00	10.42

**Table 3:** Final causes of death in the three geographical groups (number, percentage and rate/1000 live births).

**Final Causes**									
	**Metropolitan**			**Cities and Towns**			**Rural**		
	**Number**	**%**	**Rate**	**Number**	**%**	**Rate**	**Number**	**%**	**Rate**

Hypoxia	462	36.46	2.30	515	33.12	3.50	405	37.02	3.85
Immaturity related	324	25.57	1.61	642	41.29	4.37	408	37.29	3.88
Infection	175	13.81	0.87	163	10.48	1.11	93	8.50	0.88
Congenital abnormalities	209	16.50	1.04	133	8.55	0.90	85	7.77	0.81
Trauma	13	1.03	0.06	11	0.71	0.07	11	1.01	0.10
Other	84	6.63	0.42	91	5.85	0.62	92	8.41	0.87

Total	1267	100.00	6.31	1555	100.00	10.58	1094	100.00	10.40

The top ten modifiable factors associated with death in the first week are given in Tables 4, 5 and 6 for metropolitan, city and town, and rural areas respectively. The most commonly identified modifiable factors could be grouped into poor care in labour, inadequate staffing and facilities, poor neonatal care, and patient difficulties in accessing care. In metropolitan areas, the most frequent problems were inadequate foetal monitoring in labour, insufficient staff, a delay in women seeking medical help in labour followed by poor management of the second stage of labour, inadequate neonatal care facilities and insufficient transport to move patients for tertiary care. In cities and towns the major problems were a delay in women seeking medical attention in labour, inadequate antenatal care, poor intrapartum foetal monitoring followed by inadequate facilities for neonatal care, a delay in medical personnel calling for assistance when needed, and a delay in referring patients for secondary or tertiary care from primary care clinics. In rural areas the most common problems were inadequate facilities for neonatal care, a lack of antenatal care, and poor intrapartum foetal monitoring followed by a delay in women seeking care in labour, and poor management in the second stage of labour.

**Table 4:** The top ten modifiable factors in deaths in metropolitan areas.

Description	Number	% of total deaths
Poor intrapartum foetal monitoring	49	3.8
Insufficient nurses/doctors on duty to manage the patient adequately	38	3.0
Patient delay in seeking medical attention during labour	28	2.2
Prolonged 2nd stage with no intervention	24	1.9
Inadequate facilities/equipment in neonatal unit/nursery	19	1.5
Lack of institution to institution transport	19	1.5
Inadequate monitoring of the newborn infant	14	1.1
Never initiated antenatal care	11	0.9
Delay in referring patient for secondary/tertiary treatment	10	0.8
No response to apparent post-term pregnancy	8	0.6

**Table 5:** The top ten modifiable factors in deaths in cities and towns.

Description	Number	% of total deaths
Patient delay in seeking medical attention during labour	84	5.3
No or infrequent antenatal care	60	3.8
Poor intrapartum foetal monitoring	58	3.7
Inadequate facilities/equipment in neonatal unit/nursery	30	1.9
Delay in medical personnel calling for expert assistance	28	1.8
Delay in referring patient for secondary/tertiary treatment	26	1.7
Prolonged 2nd stage with no intervention	16	1
Neonatal management plan inadequate	16	1
Poor progress in labour and partogram not used correctly	16	1
Medical personnel underestimated foetal size	13	1

**Table 6:** The top ten modifiable factors in deaths in rural areas.

Description	Number	% of total deaths
Inadequate facilities/equipment in neonatal unit/nursery	44	4.0
No or poor antenatal care	39	3.5
Poor intrapartum foetal monitoring	35	3.2
Patient delay in seeking medical attention during labour	27	2.4
Prolonged 2nd stage with no intervention	16	1.4
Inappropriate response to rupture of membranes	13	1.2
Lack of home to institution transport	13	1.2
No accessible neonatal ICU bed with ventilator	10	0.9
Poor progress in labour and partogram not used correctly	10	0.9
Delay in medical personnel calling for expert assistance	9	0.8
Neonatal management plan inadequate	9	0.8

## Discussion

This is the first attempt to systematically document and identify the factors related to early neonatal deaths in South Africa. It is not an epidemiological study representing the whole country but rather an attempt to improve the care of infants in hospitals and clinics by identifying the main causes of death and common modifiable factors. It also only includes births in public hospitals and clinics willing to participate in the project. However, the numbers of births and deaths are substantial and the data were collected prospectively from both urban and rural sites after each death was discussed at a perinatal audit meeting. The findings are not surprising and probably apply to many other under resourced countries, especially in southern Africa. The higher percentage of unidentified caused of death in rural areas probably reflects a lack of clinical skills and knowledge.

The two most important causes of death were "intrapartum asphyxia and birth trauma" (intrapartum hypoxia) which resulted in neonatal "hypoxia", and "spontaneous preterm labour" leading to "immaturity related" births. The number of deaths coded as being due to the latter would have been even higher if infants weighing 500 to 999 g at birth were included. Death rates in preterm infants were particularly high in cities and towns and rural areas where neonatal high care facilities are very limited. The large number of deaths associated with perinatal hypoxia in all three groups suggested problems and inadequacies in care of women in labour and the resuscitation of newborn infants. Many of these deficiencies were identified as modifiable factors in all regions.

Almost half of the neonatal deaths due to "intrapartum asphyxia and trauma" were thought to be probably preventable. The commonest areas of suboptimal care were in foetal monitoring, monitoring the progress of labour and in managing the second stage of labour. Protocols for managing all aspects of labour are widely available and the appropriate use of partograms is strongly encouraged. The high recording rate of suboptimal care in labour is probably an indication that the knowledge on how to manage labour correctly is available, but not being adequately applied. It is disturbing that the mortality rate of term infants due to intrapartum hypoxia is so high. These deaths should be prevented and efforts to improve labour management must be intensified.

Relatively few modifiable factors were recorded, when related to the clinical management of the newborn infant. This is surprising as the large differences in neonatal death rates between metropolitan and other areas indicate that the assessment and management of newborn infants are not being performed adequately.

Specifically, only a few infants were recorded as having died as a result of poor neonatal resuscitation and care. Given the large number of neonatal deaths due to "hypoxia", it is inconceivable that this is a true reflection of the actual circumstances. There is probably poor insight into the deficiencies in the basic management of newborn infants as well as a lack of knowledge on neonatal resuscitation and care compared to intrapartum care. Time allocated to neonatal care during the training of both medical and nursing students is very limited in South Africa. If neonatal care is to be improved, more time and attention to its teaching is urgently required. A culture of questioning the neonatal management of infants who die in the first week of life must be included in perinatal mortality meetings. This will bring aspects of poor neonatal resuscitation to the fore and also act as a vehicle for training of all health workers involved in perinatal care. Currently a project to provide training in neonatal resuscitation to all health care workers is being launched by the South African Paediatric Association. Basic neonatal resuscitation is a simple skill that can be easily taught and applied. It should be made a requirement with the appropriate registration authority that all health care workers who conduct deliveries are able to provide basic neonatal resuscitation and immediate newborn care to prevent hypoxia, hypothermia and hypoglycaemia.

Improving the survival of low birth weight infants is a particular challenge in poor countries. Even in developed countries, efforts to lower the rate of preterm labour have not been successful. Therefore, as every site conducting deliveries will be faced at some time with a woman in advanced preterm labour, emphasis must be placed on excellent basic care of these small infants, often in a climate of inadequate facilities, equipment and well trained health care workers. Kangaroo mother care provides a cheap and effective answer to many of the problems posed by low birth weight infants. Recent projects in South Africa have shown that KMC can be implemented at all levels of care [13]. However, KMC must be incorporated into a package of good neonatal care and not be viewed as a stand alone intervention. Exclusive breastfeeding, clear management protocols and referral criteria, and respiratory support with nasal continuous positive airways pressure can be effectively introduced. These measurements can contribute to save infants weighing 1500 g to 2500 g.

The surprising finding of a lower rate of low birth weight infants in rural areas is unexplained but may reflect referral patterns of high risk mothers for delivery in towns and cities or a more secure food supply in rural areas than periurban slums.

A critical staff shortage was the second most common recorded modifiable factor directly involved in the death of a newborn infant in the metropolitan areas. Adequate numbers of well-trained nurses in both maternal and newborn care is probably the greatest need if the early neonatal mortality rate is to be substantially reduced in South Africa. As standards are not available to judge adequate staffing ratios, chronic understaffing is often accepted as the norm, especially outside metropolitan areas. Basic two-year courses for large numbers of primary care nurses and midwives are urgently required. Ongoing in-service support and training are also needed. However, with the current financial constraints, only a limited form of outreach training programme could realistically be made available to help staff in rural areas. Traditional methods of continuing education, where health workers are brought to regional centres for formal teaching, are no longer practical or affordable. Distance learning courses, aimed at enabling groups of practicing midwives to manage their own continuing education, have provided an alternative approach and had a major impact on the quality of maternal and perinatal care in South Africa. The Perinatal Education Programme (PEP) has been used as a self-help method of continuing training by more than 30 000 nurses over the past ten years in South Africa [14]. A number of studies have shown that PEP can significantly improve the knowledge [15], clinical skills [16], attitudes [17] and patient care practices [18] of midwives. Courses in maternal care, newborn care, primary newborn care, perinatal HIV/AIDS, mother and baby friendly care, and birth defects are available. An additional PEP course specifically addresses the knowledge and skills needed to manage perinatal mortality audits and use these data to improve perinatal care. These courses are available on an open website . However, facilitators to promote and support distance training programmes within health care regions throughout the country are still needed. Other sources of implementing best practice, such as the WHO Reproductive Health Library [19], could also help to transfer evidence into practice.

A new project in South Africa is tackling the development of appropriate, robust and cheap wind-up equipment, such as foetal heart rate and oxygen saturation monitors, to facilitate the perinatal care of infants in under resourced areas which often do not have a reliable energy supply and technological back up. The provision of adequate health infrastructures within an integrated, regional health system, better communication, and appropriate transport to improve care in rural as well as town and city areas needs to be accelerated. Specific problems such as the early identification and management of maternal syphilis are being tackled with the use of on-site screening. With the rapidly expanding AIDS pandemic, the cry for appropriate treatment for these mothers and their infants and a well planned and strengthened perinatal service is essential to support programmes that introduce the use of antiretroviral therapy and prophylaxis. With poverty alleviation and education of the general public to the importance of good, early antenatal care, many of the patient related modifiable factors could be reduced. These steps can best be achieved within the broader framework of social uplifting and job creation by the government with greater community involvement in public projects to provide a better life for all.

We now know what to do to lower the first week mortality rates of infants weighing 1000 g or more in South Africa. We also know how this can be done. What is currently lacking is not the required funding but the vision and political will to translate knowledge into practice. These challenges facing perinatal care in South Africa are mirrored in many other developing countries, especially in sub-Saharan Africa. Problems identified and lessons learned should be shared with others as we strive to improve the care of women and children and achieve the Millenium Development Goal of significantly reducing the number of childhood deaths under the age of five years in the next decade.

## Competing interests

The author(s) declare that they have no competing interests.

## Authors' contributions

Article written by R Pattinson and D Woods reviewed by D Greenfield and S Velaphi. All authors played an active role in collecting and analysing the data.
